# Functional implications of long non-coding RNAs in the pancreatic islets of Langerhans

**DOI:** 10.3389/fgene.2014.00209

**Published:** 2014-07-07

**Authors:** Jonathan L. S. Esguerra, Lena Eliasson

**Affiliations:** Islet Cell Exocytosis, Department of Clinical Sciences-Malmö, Lund University Diabetes Centre, Lund UniversityMalmö, Sweden

**Keywords:** pancreatic islets, beta-cells, insulin, glucagon, long non-coding RNA, type-2 diabetes, primate-specific

## Abstract

Type-2 diabetes (T2D) is a complex disease characterized by insulin resistance in target tissues and impaired insulin release from pancreatic beta cells. As central tissue of glucose homeostasis, the pancreatic islet continues to be an important focus of research to understand the pathophysiology of the disease. The increased access to human pancreatic islets has resulted in improved knowledge of islet function, and together with advances in RNA sequencing and related technologies, revealed the transcriptional and epigenetic landscape of human islet cells. The discovery of thousands of long non-coding RNA (lncRNA) transcripts highly enriched in the pancreatic islet and/or specifically expressed in the beta-cells, points to yet another layer of gene regulation of many hitherto unknown mechanistic principles governing islet cell functions. Here we review fundamental islet physiology and propose functional implications of the lncRNAs in islet development and endocrine cell functions. We also take into account important differences between rodent and human islets in terms of morphology and function, and suggest how species-specific lncRNAs may partly influence gene regulation to define the unique phenotypic identity of an organism and the functions of its constituent cells. The implication of primate-specific lncRNAs will be far-reaching in all aspects of diabetes research, but most importantly in the identification and development of novel targets to improve pancreatic islet cell functions as a therapeutic approach to treat T2D.

## INTRODUCTION

The field of pancreatic islet research has largely benefited from the use of animal models, particularly in the investigation of molecular processes governing islet development and functions using rodents. While many features of rodent islets have been observed to reflect those found in humans, the increasing availability of human pancreatic islets for basic cell physiological and histological research has made it evident that important differences exist in terms of islet architecture (Brissova et al., 2005; Cabrera et al., 2006; Fujita et al., 2011; Wang et al., 2013), and islet cell functions (Ashcroft and Rorsman, 2012; Rorsman and Braun, 2013; Gylfe and Gilon, 2014). Moreover advances in RNA sequencing (RNA-seq) and complementary “transcriptome annotation” technologies enabled the exploration of the transcriptional and epigenetic landscape of human islet cells in unprecedented resolution revealing both short and long non-coding RNAs (lncRNAs) important for islet function (Eliasson and Esguerra, 2014). Here we will focus on lncRNA transcripts in pancreatic islets. The presence of lncRNAs highly enriched in the pancreatic islet and/or specifically expressed in the beta-cells provides a rich source of novel modes of gene regulation governing islet functions. Moreover, recent studies suggest the presence of a large proportion of primate-specific lncRNAs (Derrien et al., 2012). While evolutionary conservation of gene loci across broad phyla strongly points to a functional role of corresponding gene products, and hence more likely contribute to the phenotype, we would like to argue that species-specific transcripts play a significant role in defining the unique phenotypic identity. Currently however, experimental data that links primate-specific lncRNAs to the human islet phenotype are limited. It will be a daunting task to evaluate the connection between primate-specific lncRNAs and human islet specific protein expression. In this review we try to answer the questions: (1) what are the potential roles of lncRNAs in islet development and endocrine cell function and, (2) can we attribute many of the observed islet morphological and functional differences in different species on non-evolutionary-conserved lncRNAs?

## PANCREATIC ISLETS OF LANGERHANS AND TYPE-2 DIABETES

The islets of Langerhans in the pancreas are central to carbohydrate homeostasis in higher metazoans. In vertebrates, the islets are composed primarily of alpha and beta cells which secrete glucagon and insulin, respectively. Glucagon triggers glycogenolysis in the liver where glycogen reserves are converted into glucose prior to release into the blood stream. Glucagon also mediates control of glucose production by triggering the phosphorylation of key enzymes that either inhibit glucose-requiring glycolysis or stimulate gluconeogenesis (Gromada et al., 2007). Hence, glucagon is secreted during periods of hypoglycemia when blood glucose levels are low, such as during starvation, fasting or exercise. Patients with T2D have increased glucagon secretion that exaggerates the disease state, and it is suggested that dysfunctional glucagon is due to impaired intrinsic glucose regulation (Rorsman et al., 2008) and/or loss of the characteristic phasic relationship between insulin and glucagon secretion (Gylfe and Gilon, 2014).

The effect of the insulin hormone is counter-regulatory to glucagon. Hyperglycemic conditions stimulate the beta cells to release insulin into the blood stream. This promotes glucose uptake into target tissues, e.g., fat and muscle, resulting in decreased blood glucose levels. Insulin resistance therefore refers to a pathophysiological condition when tissues fail to respond to normal insulin levels, thereby triggering an adaptive compensatory response from the beta cells to release more insulin (Prentki and Nolan, 2006). While such compensatory beta cell adaptations may provide short-term relief, the long term consequence to the beta cells is deleterious: beginning with impaired insulin secretion capacity to outright beta cell failure as diabetes progresses.

Evidence so far point to combined effects of reduced beta-cell mass and impaired beta-cell function as primary drivers of T2D development (Meier and Bonadonna, 2013). Previous studies using immunohistochemical methods showed reduced beta cell mass in human T2D islets due to apoptosis (Butler et al., 2003), although lineage tracing of FoxO1-deficient beta cells in mice suggests that such beta cell mass reduction could also be due to dedifferentiation of the beta cells into alpha cells (Talchai et al., 2012). Our recent ultrastructural analyses of electromicroscopic images from human islet preparations however, indicate that beta cell mass is not significantly reduced in human T2D islets, implying that the pathogenesis lies primarily in impaired beta cell function, e.g., defective insulin production and/or secretion (Dayeh et al., 2014), which could be partly attributed to reduced expression of key beta cell-specific transcriptional regulators including MAFA, NKX6.1, and PDX1 (Guo et al., 2013). Indeed, many of the genes in the vicinity of single nucleotide polymorphisms (SNPs), which could be linked to predisposition to T2D, are usually involved in pancreatic beta cell functions (Groop and Lyssenko, 2009; Rosengren et al., 2012). Moreover, epigenetic changes in islets from T2D patients correlated with expression of genes involved in insulin secretion (Dayeh et al., 2014).

### COMPARATIVE ANATOMY OF HUMAN AND RODENT ISLETS

A striking feature of isolated islets, regardless of species they are derived from, is their coherence into a compact cluster of cells. The earliest investigations were therefore focused in elucidating the types of endocrine cells constituting the islet, and whether the different islet cell types exhibit spatial organization. Here we concentrate on the main cytoarchitectural features of rodents (rat and mice) and human islets. A comprehensive treatise on islet comparative anatomy among different taxonomic groups from ancient fish to primates may be found elsewhere (Heller, 2010).

**FIGURE 1:**
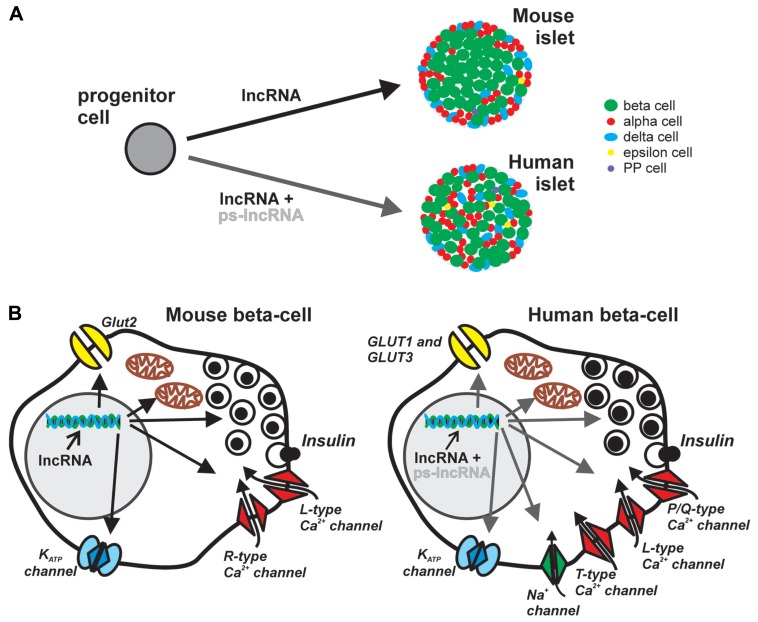
**Potential roles of lncRNA in pancreatic islet development and endocrine cell function.** Majority of lncRNAs are localized in the nucleus and participate in transcriptional and epigenetic regulation, acting as scaffolds of transcription and chromatin-modifying factors, or as transcriptional enhancers. **(A)** Regardless of species, different lncRNAs may influence the cell-fate and maturation of the various endocrine cell types. However, the cytoarchitectural differences, i.e., islet cellular composition, proportion, organization, innervations and vascularization patterns, between mouse and human islets may be partly determined by species-specific lncRNAs such as the large cluster of primate-specific (ps)-lncRNAs discovered in human cells. **(B)** The role of lncRNAs in endocrine islet cell functions, here represented by the components of canonical stimulus-secretion coupling in mouse (left) and human (right) beta cells, may also be in the level of transcriptional and epigenetic regulation of factors involved in cell-type specific functions. Considerable differences in nutrient sensing, metabolism, exocytotic events and electrophysiological properties between human and mouse beta cells may be a consequence of differential regulation of relevant genes, e.g., ion-channels and exocytotic proteins, by ps-lncRNAs.

Compared with rodent islets, there is considerable heterogeneity in human islets in two levels: (i) islet cellular composition, proportion and organization, and (ii) distinction between small and large human islets, including differences in islet composition depending on their regional location in the pancreas (Brissova et al., 2005; Cabrera et al., 2006; Fujita et al., 2011; Wang et al., 2013). Mouse islets comprise up to 75% beta cells mainly in the core and surrounded by a mantel of ∼20% alpha cells, and ∼5% delta (somatostatin) cells (Brissova et al., 2005). In contrast, human islets have more scattered, random-like arrangement of the different islet cell types, with many beta cells also prominently located on the outer periphery (**Figure 1A**). On average, human islets contain ∼55% beta cells, ∼35% alpha cells, and ∼10% delta cells (Brissova et al., 2005). There are also other hormone-producing cells in the islets such as the PP (polypeptide) and ghrelin-producing epsilon cells identified in humans, rodents, and several mammals. Interestingly, only the adult human islets harbor a substantial number of ghrelin-producing epsilon cells (Wierup et al., 2014). During embryonic development in certain mammalian species, transient expression of serotonin-producing enterochromaffin cells (Alumets et al., 1983) and gastrin-producing G-cells have also been demonstrated in the pancreas (Suissa et al., 2013). Thus there are at least seven hormone-secreting endocrine cell types identified in the islets of different species at some point of pancreatic development (Wierup et al., 2014). It is worth mentioning that of all the studied model animals, only the non-human primates are found to have very similar islet cell distribution and organization as in humans (Brissova et al., 2005). This could be reflected by the high genetic similarities within the primate clade as shown in a comprehensive review on comparative genomics of human and more than a dozen non-human primates (Rogers and Gibbs, 2014).

The parasympathetic and sympathetic innervation patterns have also been shown to be very different in the human islets, with considerable implication in the autonomic control of hormone secretion. The human endocrine cells, as opposed to mouse endocrine cells, have fewer contacts with autonomic axons, which means less direct autonomic control of endocrine cell functions (Rodriguez-Diaz et al., 2011a). Instead smooth muscle cells of human islet blood vessels are found to be innervated with sympathetic fibers, with the implication that hormone secretion may be modulated by local blood flow (Rodriguez-Diaz et al., 2011a). Moreover the sparse cholinergic innervation within human islets appears to be compensated by the ability of the human alpha cells to secrete acetylcholine, which provides paracrine signal to the beta cells in response to impending increase in glucose concentration (Rodriguez-Diaz et al., 2011b).

While the many similarities between human and rodent islets have allowed the dissection of important shared biological processes in islet development and function, emerging findings on species-specific differences on pancreatic islet organization and composition, specifically between primates and other mammalian clades, highlight fundamental differences by which glucose homeostasis may be controlled. Indeed, the clinical and pathological features in non-human primate models of T2D impeccably reflect those of human T2D, making the translation of novel therapeutic agents from non-human primates to humans highly predictive (Hansen, 2012; Harwood et al., 2012). Corollary to such evidence, utmost caution must be exercised when extrapolating findings in rodent models to man when it comes to control of islet development, assessment of islet quality, insulin secretion capacity, and other techniques which may be confounded by the three-dimensional arrangement of the different endocrine cell types in the islets (Brissova et al., 2005).

### FUNCTIONAL DIFFERENCES BETWEEN HUMAN AND RODENT ISLETS

An obvious consequence of cytoarchitectural/morphological differences between human and rodent islets is differences in the relative accessibility of the different endocrine cell types from nutrient and paracrine signals. The unique innervation and vascularization patterns, combined with the apparent random organization of the various endocrine cells in the human islets allow for more contact of the cells with the environment, and closer interactions between the different islet cell types resulting in more enhanced paracrine signaling (Cabrera et al., 2006). The effect of endogenous hormones secreted by the different islet cell types on one another is not trivial; ghrelin predominately inhibits insulin secretion (Wierup et al., 2014), somatostatin inhibits both insulin and glucagon release (Strowski et al., 2000), and both glucagon and insulin influence each other’s secretion (Gromada et al., 2007; Bansal and Wang, 2008). Indeed, the significance of the interplay between functional alpha- and beta cell regulation in the pathogenesis of diabetes was highlighted in a recent review (Unger and Cherrington, 2012), suggesting a major role for dysfunctional glucagon release in the disease development.

Studies of whole islet physiology reveal subtle but important differences between murine and human islets. Even between mouse and rat islets, difference in insulin secretion in response to certain metabolites has been demonstrated. For instance, the absence of malic enzyme in mouse beta cells renders the cells unresponsive to dimethylsuccinate, in contrast to rat beta cells (MacDonald, 2002). While nutrient-induced insulin secretion was found to be globally similar between rodent and human islets, with the presence of both triggering and amplifying pathways, the concentration-response curve is shifted to the left in humans, compatible with the observation that humans have generally lower plasma glucose levels than rodents (Henquin et al., 2006).

The exact molecular mechanisms underlying discrepancies in different species regarding the response of islet cells on nutrient stimulation are not entirely known. However, differences in the major components of the stimulus-secretion coupling (**Figure 1B**) may provide important clues. In human beta cells, the main glucose transporters GLUT-1 and GLUT-3 have considerably higher affinity for the substrate as exemplified by their lower *K*_m_ values, 6 and 1 mM, respectively than the main glucose transporter, Glut2, in rodents with *K*_m_ of 11 mM (McCulloch et al., 2011; Rorsman and Braun, 2013). Another aspect of stimulus-secretion coupling is the electrically excitable nature of beta cells which highlights the importance of ion channels in generating action potentials leading to insulin secretion. The *K*_ATP_ channel controls the electrical activity in both mouse and human beta cells, but the resting membrane conductance at 1 mM glucose is ten times lower in human beta cell, contributing to initiation of insulin secretion at low glucose levels (5–6 mM; Rorsman and Braun, 2013). In addition, human beta cells are equipped with T-type Ca^2+^ channels that are absent in mice. Together with current flow through voltage- dependent Na^+^ channel these channels contribute to the upstroke of the action potential in human beta-cells. (Barnett et al., 1995; Braun et al., 2008). The final increase in Ca^2+^ triggering exocytosis of insulin granules arise from influx through L-type and P/Q-type Ca^2+^ channels in rodent and human beta cells, respectively (Barg et al., 2001; Braun et al., 2009). The revelation of several electrophysiological differences between rodent and human beta cells (Rorsman and Braun, 2013), primarily due to differential expression, and/or transcriptional regulation by yet unknown principles [alternative splicing, microRNA (miRNA), lncRNAs], of functional ion channels underscores the importance of species-specific genetic basis of regulation of insulin secretion. More in depth characterization of human islets will further reveal such species differences in other aspects of molecular control of endocrine hormone secretion. Recently, analyses of anaplerotic products show much lower dependence of human islets to the activity of the key anaplerotic enzyme, pyruvate carboxylase (MacDonald et al., 2011). Finally, in the search for exocytotic genes differentially expressed in human T2D islets, synaptotagmin isoforms previously deemed unimportant in mice beta cell exocytosis, correlated with insulin secretion in humans (Andersson et al., 2012).

To understand the origin of morphological and functional differences in rodent and human pancreatic islets, one has to consider the developmental aspect of endocrine cell differentiation (**Figure 1A**). Conventional wisdom dictates that regardless of species, the path from endoderm progenitors to differentiated hormone-secreting cells of the pancreatic islets is a highly orchestrated process mediated by precise spatio-temporal interplay of various transcription factors. However, significant differences exist in the transcriptional repertoire of endocrine cell differentiation between human and mouse. A survey of known regulators of mouse endocrine pancreatic cell fate in purified human beta and alpha cells reveals that *MAFB* which is not expressed in adult mouse beta cells, is present in comparable levels in both human alpha and beta cells (Dorrell et al., 2011). In the same study, *IRX2* previously shown to be expressed only in the developing mouse pancreas (Petri et al., 2006), was found to persist in adult human alpha cells. Recently, the pancreas-enriched miR-7 was also found to negatively regulate Pax6 which has a central role in endocrine cell differentiation and maintenance of identity (Kredo-Russo et al., 2012). Although miR-7 is a broadly conserved miRNA, it is possible that it may also target other non-conserved mRNAs which may impart species-specific fine-tuning of regulatory circuits in the context of islet development. Indeed, both evolutionary conserved and non-conserved targets for individual miRNAs have been predicted and demonstrated (Betel et al., 2010). The involvement of non-coding RNAs in pancreatic islet development adds another level of regulation of cell fate trajectories and distinct cell-type specific functions.

## LONG NON-CODING RNAs

Long non-coding RNAs are transcripts without protein-coding potential, arbitrarily defined in size by a cut-off length of >200 nucleotides (HUGO Gene Nomenclature Committee; Seal et al., 2011). Most lncRNAs are transcribed by RNA polymerase II, and share many properties of mRNAs such as splicing, capping and polyadenylation (Derrien et al., 2012). Similar to protein-coding genes, the expression of lncRNAs is tightly regulated and display spatio-temporal expression patterns, i.e., cell-type specific and/or developmental stage-specific expression (Dinger et al., 2008; Mercer et al., 2008; Cabili et al., 2011).

Integrative analysis of RNA-seq data with other complementary high-throughput “transcript annotation” technologies, e.g., transcription initiation mapping by cap-analysis of gene expression (CAGE; Kodzius et al., 2006) and identification of sites of 5^′^ and 3^′^ transcript termini (Ng et al., 2005), reveals that lncRNAs may generally be categorized with respect to their genomic position either as “intergenic” (between protein-coding genes), or “genic” (Derrien et al., 2012). Intergenic lncRNAs or “lincRNAs” (long intergenic non-coding RNAs) are encoded as distinct transcriptional units within genomic regions which used to be called “gene deserts.” The “genic” lncRNAs may be exonic, intronic, or overlapping, and can be further classified as either in the sense or antisense strand relative to the protein-coding gene (Derrien et al., 2012). An in-depth investigation on expression dynamics of lncRNAs during differentiation of human neuroblastoma cells suggests 19 different genomic architecture classes of lncRNAs based on both their relative positions with protein-coding genes, and on the orientations of their transcription (Batagov et al., 2013).

The GENCODE (encyclopædia of genes and gene variants) project lists 13870 lncRNA genes in the human genome (Version 19, July 2013 freeze, GRCh37 – Ensembl 74) and 4074 lncRNA genes in the mouse genome (Version M2, July 2013 freeze, GRCm38 – Ensembl 74; www.gencodegenes.org/; Dunham et al., 2012). There are other independent efforts in annotating lncRNAs in the human genome, albeit with surprisingly low overlap with the GENCODE annotations. For example, only 39% of the 4662 human lincRNA loci cataloged in Cabili et al. (2011) study intersected with those of GENCODE’s human lncRNAs (Derrien et al., 2012). Thus, while there is an indisputable consensus about the widespread transcription of lncRNA genes in human and other mammalian cells, the field is still mostly in the exploratory stage, and both high-throughput biochemical data generation and *in silico* analyses warrant further development to aid in standardization of analytical procedures.

### ROLES OF lncRNA

A wide variety of functions have been attributed to lncRNAs including roles in transcriptional regulation (Penny et al., 1996; Orom et al., 2010; Santoro et al., 2013), as architectural determinants of subcellular structures (Clemson et al., 2009; Batista and Chang, 2013), in epigenetic inheritance/chromatin dynamics (Khalil et al., 2009; Tsai et al., 2010), and most recently in higher-order chromosomal organization (Hacisuleyman et al., 2014). Here we will only briefly mention the general characteristics of lncRNA mechanisms of action. A more detailed description of some well-characterized lncRNAs is described elsewhere in recent reviews (Kornfeld and Bruning, 2014; Yang et al., 2014a).

As entities of transcriptional control, it is emerging that lncRNAs may perform their functions in at least two ways: (i) as scaffoldings in ribonucleoprotein complexes, e.g., transcription or chromatin-modifying factors, acting in *cis* or in *trans* on the genome (Yang et al., 2014a), and (ii) as incidental by-products of a negative type of transcriptional regulation termed, “transcriptional interference” (Kornienko et al., 2013). Prior knowledge about the mechanism of action of a few well-characterized lncRNAs such as the HOTAIR (Rinn et al., 2007), and the observation that as many as 24% of human lincRNAs bind the polycomb repressive complex 2 (PRC2), responsible for transcriptional repression of specific genes by methylation of H3K27 (Khalil et al., 2009) strengthened the hypothesis that lncRNAs provide the specificity in guiding chromatin-modifying complexes into exact regions in the genome. Whether the guiding mechanism is based on sequence-complementarity between the lncRNA and genomic DNA, or other motif-recognition process remain to be seen (Guttman and Rinn, 2012). In a separate attempt to functionally categorize lncRNAs *en masse*, it was shown that many lncRNAs have enhancer-like properties, activating and/or potentiating the expression of neighboring protein-coding genes (Orom et al., 2010).

Recently, it was shown that a lncRNA called “functional intergenic repeating RNA element” (*Firre*), facilitates trans-chromosomal interactions, in which genes involved in energy metabolism and adipogenesis are brought in close proximity, presumably to allow efficient co-regulation of genes in the same biological pathway (Hacisuleyman et al., 2014).

In summary, lncRNAs characterized thus far display distinct functions and mode of regulation, again reminiscent of protein-coding genes belonging to broad functional ontologies. Nevertheless, as exemplified by PRC2-binding lncRNAs, or the enhancer-like lncRNAs, consensus mechanisms of action are emerging for many lncRNAs which may permit their classification into specific functional categories.

### EVOLUTIONARY CONSERVATION OF lncRNAs

Previous experience from large-scale, computational prediction of protein-coding genes as crucial first step in de novo genome annotation, showed that DNA sequence conservation across broad phyla is a good indicator of genuine coding potential, i.e., that the gene will produce an mRNA transcript and will eventually be translated, and hence has biologically meaningful functions. Although lncRNAs have been experimentally discovered from transcription data, it is still deemed necessary to use the criterion of evolutionary conservation to help distinguish functional RNA transcripts from transcriptional and experimental noise. However, sequence conservation alone is inherently problematic for non-coding RNA genes whose functional gene products act in the level of secondary or tertiary structural RNA features. Formation of secondary RNA structures are not evolutionary constrained to maintain nucleotide sequences in the same way that protein-coding genes are constrained to maintain specific codon sequences to ensure functional proteins. For instance, RNA hairpin loops which are ubiquitous secondary structural feature of virtually all functional non-coding RNA molecules may be formed irrespective of the nucleotide sequence, as long as energetically favorable base-pairings in the hairpin stem are maintained. It is perhaps not surprising that when standard sequence alignment procedures are used to assess the conservation of lncRNAs in various species, consistently modest sequence similarity is found. Indeed, compared to protein-coding sequences aligned between different species, much lower sequence identities are found for each of the 993 syntenically paired orthologous lincRNAs in mammals and other vertebrates (Cabili et al., 2011). However, the promoter regions of the lncRNAs are shown to be more conserved than the exonic regions which imply similar regulation and potentially analogous roles of orthologous lncRNAs in different species (Guttman et al., 2009; Derrien et al., 2012).

Remarkably, despite employing meticulous approaches in aligning genomes, one study finds only 12% (993 lincRNAs) of human lincRNAs with orthologous sequences in another vertebrate species (Cabili et al., 2011), while GENCODE v.7 reports 30% of all annotated lncRNAs, ∼4500 lncRNAs, to be clearly primate-specific (Derrien et al., 2012). The manually curated lncRNA database (www.lncrnadb.org) lists a number of primate-specific lncRNAs (Amaral et al., 2011), including the 482-nucleotide long HULC RNA found to be highly upregulated in human hepatocellular carcinoma (Panzitt et al., 2007). One recently investigated human-specific non-coding RNA, miR-941, is expressed in the brain and regulates genes involved in neurotransmitter signaling (Hu et al., 2012). Thus, conservation of gene locus, let alone gene sequence across broad phyla is not a strict requirement for biological function. After all, it is the evolution of genetic differences that ultimately drives speciation. The non-conserved lncRNAs must be bona fide elements of genetic programs which specify phenotypic/morphological differences between organisms.

## EXPRESSION OF lncRNAs IN HUMAN PANCREATIC ISLET CELLS

By integrating transcriptomics data and chromatin maps, 1128 lncRNAs were reliably identified in purified human pancreatic islets (Moran et al., 2012). Many of the lncRNAs were specifically expressed in the islets and beta cells, suggesting important roles in the developmental programming, proper functioning and/or maintenance of the pancreatic endocrine tissue. Indeed, the expression levels of a dozen lncRNAs were found to fluctuate during stage-specific embryonic stem cell differentiation relative to the final expression in *in vivo* functional endocrine cells, and at least two lncRNAs, HI-LNC78 and HI-LNC80, exhibited dynamic upregulation when the islets were exposed to high glucose concentrations (Moran et al., 2012). In a separate deep RNA-seq study of purified human beta cells, 148 lincRNAs were found to be overexpressed in beta cells compared to non-beta cells (Nica et al., 2013), while another study discovered 12 beta cell-specific and 5 alpha cell-specific lncRNAs (Bramswig et al., 2013). Taken together, these findings suggest the importance of cell-type- and/or condition-specific expression of lncRNAs in the human pancreatic islet.

All the aforementioned studies on lncRNAs in the human pancreatic islets are exploratory in nature, and no particular mechanism of action has been attributed so far on any of the identified lncRNAs in the islet cells. Elucidating the molecular functions of islet cell-specific or – enriched lncRNAs will be challenging because of the generally low conservation of lncRNAs in commonly used rodent and *in vitro* models. Indeed, RNA-seq of mouse pancreatic beta cell transcriptome corroborates previous findings about the very weak conservation of lincRNAs in humans (Ku et al., 2012), although it was shown that for a considerable number of lincRNAs, short conserved stretches of sequences may be enough to guarantee conserved function in vertebrate embryonic development (Ulitsky et al., 2011). In view of species-specific expression of many lncRNAs, the recently developed human beta cell lines from Ravassard et al. (2011), EndoC-BH1 and EndoC-BH2 (Scharfmann et al., 2014) will be valuable in dissecting the molecular functions of lncRNAs in the beta cell.

### POTENTIAL ROLES OF lncRNAs IN THE DEVELOPMENT, FUNCTION AND MAINTENANCE OF PANCREATIC ISLET CELLS: ACQUISITION OF SPECIES-SPECIFIC ISLET PHENOTYPE/MORPHOLOGY AND CELL-TYPE SPECIFIC FUNCTIONS

In light of scarce experimental data on recently discovered lncRNAs in islet cells, we can only infer about their potential molecular roles based on findings in other cell types. The various mechanisms by which lncRNAs were shown to exert their functions will undoubtedly also influence pancreatic islet cells in terms of cellular differentiation and development, specifically in maintaining cellular identity and plasticity. They may also be important component of islet cells stress response, such as in activating beta cell compensatory mechanisms in countering environmental stressors in T2D. The role of lncRNAs in the acquisition of species-specific islet phenotype/morphology, and maintenance of cellular phenotype of pancreatic islet cells may be operational in two levels: (i) between islet cell types of different species, and (ii) among the islet cells of the same species.

Almost a third of human lncRNAs (∼330 lncRNAs) discovered in the human islets lack orthologous sequences in mice (Moran et al., 2012). Given the tendency of many lncRNAs to associate with components of chromatin-modifying complexes with roles in embryonic stem cell fates (Dinger et al., 2008; Yang et al., 2014b), the involvement of primate-specific lncRNA in the developmental program of pancreatic islets is possible, and may (i) have direct contribution to the origin of cytoarchitectural differences between islets in the different species (**Figure 1A**), and (ii) contribute to the differential expression of essential proteins in islet cell secretion (**Figure 1B**). In the same line of reasoning, a number of islet cell-type specific lncRNAs recently reported (12 beta cell-specific and 5 alpha cell-specific; Bramswig et al., 2013) could play essential roles in conferring cell-type specific functions in the pancreaticislets.

The islet cells are constantly subjected to fluctuating nutrient stimuli and are challenged to respond accordingly to maintain glucose homeostasis. The coordinated transcription of many genes required to overcome this challenge relies on transcription factors activating specific sets of genes. Indeed, rat islets subjected to different glucose concentrations showed distinct clusters of mRNA profiles suggesting highly coordinated response to varying nutrient stimuli (Bensellam et al., 2009). It will be interesting whether *Firre-*like lncRNAs involved in trans-chromosomal interactions (Hacisuleyman et al., 2014) are present in endocrine cells to facilitate regulation of cell-type specific pathways by acting as scaffolds guiding transcription factors to target genes. Many of the identified human islet lncRNAs lie adjacent to islet-specific chromatin domains and protein-coding genes (Moran et al., 2012), a striking example being *HI-LNC25* whose closest neighbor is *MAFB* which is a regulator of islet-cell maturation (Artner et al., 2007) and as was discussed earlier absent in adult mouse beta cells, but present in both human alpha and beta cells (Dorrell et al., 2011).

Enhancer elements are also key determinants of islet-specific gene activity (Pasquali et al., 2014). Notably, Cabili et al. (2011) report that 27% (∼1200) of human lincRNAs overlap with known enhancer regions in the genome. It will be interesting to examine the presence of lncRNAs in human pancreatic islet enhancer clusters reported to be enriched in T2D risk-associated variants (Pasquali et al., 2014).

Extensive mapping of the epigenome of whole human pancreatic islets (Bhandare et al., 2010; Gaulton et al., 2010; Stitzel et al., 2010; Dayeh et al., 2014), and of purified islet cells (Bramswig et al., 2013; Dayeh et al., 2014), reveal cell-type specific epigenetic landscape delineating sites of active/inactive gene transcription. The majority of lncRNAs shown to associate with various chromatin-modifying factors are tantalizing candidate factors which could provide additional molecular specificity in targeting epigenetic markers in the pancreatic islet cells. It is tempting to hypothesize that primate-specific lncRNAs could be involved in specifying the expression of certain components of the stimulus-secretion coupling in the beta cells, which in turn potentially contributes in species-specific response of beta cells to nutrient stimuli (**Figure 1B**).

## CONCLUSION

The pervasive nature of eukaryotic gene transcription revealed by next-generation sequencing and associated technologies for mapping transcriptional activity brought into the limelight a plethora of non-coding RNA classes of hitherto unknown functions. The existence of long functional RNA molecules provides tantalizing hypotheses on how chromatin-modifying and transcription factors may act upon their genomic targets in a highly loci-specific recognition process. This provides an important mechanistic insight into how specificity is achieved by various factors in pancreatic islets cells to coordinate the regulation of multiple genes responsible for cell-type specific phenotypes and functions.

The potential roles of lncRNAs in pancreatic islet development, specifically in endocrine cell-fate determination and subsequent maintenance of cellular identities and functions, will broaden our understanding of pancreatic islets, and hence open up new possibilities in identifying novel therapeutic targets in treating type-2 diabetes (T2D). However, the implication of numerous primate-specific lncRNAs will be a particular challenge in the field which heavily depends on rodent models when trying to elucidate the molecular basis of metabolic pathophysiologies. In particular, considering the complex ethical issues involved, the *in vivo* roles of primate-specific lncRNAs will pose nagging questions in many years to come.

## Conflict of Interest Statement

The authors declare that the research was conducted in the absence of any commercial or financial relationships that could be construed as a potential conflict of interest.
